# Cadherin-11 Regulates Motility in Normal Cortical Neural Precursors and Glioblastoma

**DOI:** 10.1371/journal.pone.0070962

**Published:** 2013-08-07

**Authors:** Jessica D. Schulte, Maya Srikanth, Sunit Das, Jianing Zhang, Justin D. Lathia, Lihui Yin, Jeremy N. Rich, Eric C. Olson, John A. Kessler, Anjen Chenn

**Affiliations:** 1 Department of Pathology, Feinberg School of Medicine, Northwestern University, Chicago, Illinois, United States of America; 2 Davee Department of Neurology, Feinberg School of Medicine, Northwestern University, Chicago, Illinois, United States of America; 3 Department of Cellular and Molecular Medicine, Lerner Research Institute, Cleveland Clinic, Cleveland, Ohio, United States of America; 4 Department of Stem Cell Biology and Regenerative Medicine, Lerner Research Institute, Cleveland Clinic, Cleveland, Ohio, United States of America; 5 Department of Neuroscience and Physiology, State University of New York, Upstate. Syracuse, New York, United States of America; Northwestern University Feinberg School of Medicine, United States of America

## Abstract

Metastasizing tumor cells undergo a transformation that resembles a process in normal development when non-migratory epithelial cells modulate the expression of cytoskeletal and adhesion proteins to promote cell motility. Here we find a mesenchymal cadherin, Cadherin-11 (CDH11), is increased in cells exiting the ventricular zone (VZ) neuroepithelium during normal cerebral cortical development. When overexpressed in cortical progenitors *in vivo*, CDH11 causes premature exit from the neuroepithelium and increased cell migration. CDH11 expression is elevated in human brain tumors, correlating with higher tumor grade and decreased patient survival. In glioblastoma, CDH11-expressing tumor cells can be found localized near tumor vasculature. Endothelial cells stimulate TGFβ signaling and CDH11 expression in glioblastoma cells. TGFβ promotes glioblastoma cell motility, and knockdown of CDH11 expression in primary human glioblastoma cells inhibits TGFβ-stimulated migration. Together, these findings show that Cadherin-11 can promote cell migration in neural precursors and glioblastoma cells and suggest that endothelial cells increase tumor aggressiveness by co-opting mechanisms that regulate normal neural development.

## Introduction

Mechanisms that regulate normal development can be co-opted in tumor development. Phenotypic and molecular alterations in cancer cells resembling the epithelial to mesenchymal transitions (EMT) that occur during normal organ development have been suggested as a critical step in the metastatic cascade [1]–[3]. As varying degrees of EMT correlate with invasiveness and poor prognosis of multiple human cancers, a greater understanding of the factors regulating EMT may lead to potential therapeutic approaches in cancer [4]. Glioblastoma (GBM) is the most common and aggressive primary brain tumor in adults. GBM cells are highly migratory and extensive infiltration of GBM cells into brain parenchyma renders curative surgical resection virtually impossible [5]. Here we examine whether the EMT-like processes occurring in normal neural development may also regulate GBM cell migration.

In normal cerebral cortical development, cortical neurons are produced from a population of proliferating precursor cells in the ventricular zone (VZ), a neuropithelial progenitor zone that lines the lateral ventricles of the developing brain. Electron micrographs [6], [7] and immunohistological characterization [8] indicate that the apical endfeet of cortical progenitors are joined to each other by adherens junctions, enriched for a number of signaling molecules, including αE-catenin, β-catenin, N-cadherin, pp120, and paxillin [8], [9].

**Figure 1:**
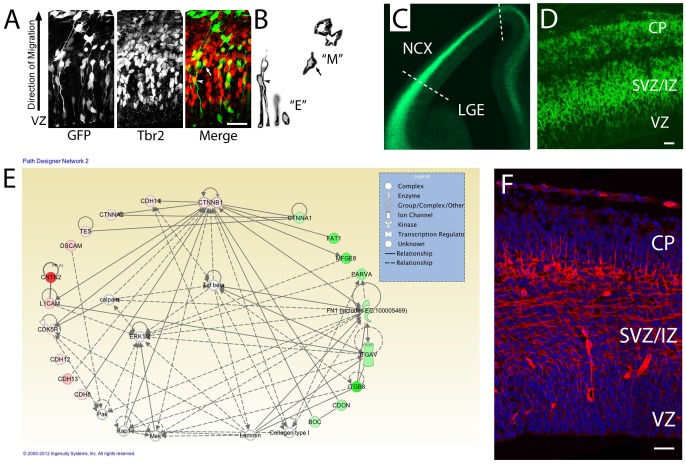
Network analysis of dynamically-regulated adhesion molecules reveal that cortical cells express Cdh11 as they transition from polarized epithelial to multipolar migratory cells and exit the VZ. (**A**) VZ cells electroporated with GFP expression plasmid reveal both polarized epithelial morphology “E” and multipolar migratory morphology “M” in the developing cortex. Immunostaining for the intermediate progenitor marker Tbr2 (Eomes) highlight cells undergoing the transition from E (arrowhead) to M (arrow). (**B**) Images extracted from the GFP image in (**A**) showing cells with epithelial “E” and multipolar “M” morphology. (**C, D**) Section of E12.5 Eomes:GFP reporter mouse. Dotted lines show how the cortex was separated, then dissociated into single cells for FACS. (**D**) Higher power image through E12.5 Eomes:GFP reporter mouse. (**E**) >3-fold up and >3-fold down regulated adhesion molecules were imported with β-catenin to populate the IPA network. Green colors indicate genes with higher expression in VZ and red indicate higher expression outside VZ. Solid lines indicate direct protein-protein interactions. (F) Immunoreactivity for CDH11 in E14.5 mouse cerebral cortex showing enriched expression in SVZ/IZ and deeper CP. Bar = 50 µm.

Differentiating cortical progenitors disassemble their adherens junctions and undergo a transition from polarized neuroepithelial cells to multipolar migratory cells as they exit the VZ (**Fig. 1** **A, B**). In addition to the morphological changes that occur when precursor cells exit the VZ, cellular migration is coupled with changes in the expression of characteristic molecular markers, such as the downregulation of the Pax6 transcription factor, the Nestin intermediate filament, the adherens junction protein αE-catenin, and upregulation of the TBR2 and INSM1 transcription factors, and ultimately, as cells differentiate into neurons, expression of neuron-specific class III β-tubulin (TuJ1) and a neuron-specific microtubule-binding protein doublecortin (DCX) [9], [10]. The mechanisms regulating the initiation of migration from the VZ neuroepithelium are poorly understood, and whether similar mechanisms regulate GBM cell motility are unclear.

## Materials and Methods

### Ethics Statement

Glioma stem cells (GSCs) were isolated from GBM primary surgical specimens in keeping with protocols approved by the Northwestern University Institutional Review Board, NU 07C2: “Lineage determination of brain tumor stem-like cells (BTSCs) harvested from human astrocytic and oligodendroglial tumors.” Written informed consent was obtained from the donors or next of kin for the use of tissue samples for this research. All mice in this study were treated according to protocols reviewed and approved by the institutional animal care and use committees of Northwestern University, Animal Study Protocol # 2010-1863, approved by the Northwestern University Office for the Protection of Research Subjects Institutional Animal Care and Use Commitee.

### Animals

Timed-pregnant C57BL/6 mice were ordered from Charles River Laboratories (Wilmington, MA). The genes differentially-regulated as cells exit the VZ were previously identified using fluorescence activated cell sorting (FACS), from GFP-expressing cells from embryonic E14.5 Eomes:: GFP transgenic mice [11]. Tg(Eomes::eGFP)Gsat mice (The Gene Expression Nervous System Atlas Project, NINDS Contract # N01NS02331 to The Rockefeller University, New York, NY) were mated to C57Bl6 (Taconic Labs, Germantown, NY) to generate embryos. The day of plug discovery was designated embryonic day 0.5 (E0.5).

### *In Utero* electroporation

For *in utero* injection, timed-pregnant C57Bl6 mice at E13.5 were anesthetized using inhalation of isoflurane mixed in a constant ratio with oxygen, after which abdominal fur was removed, and the uterine horns were exposed through a midline laparotomy incision. DNA solution (2.5 µL, 0.25 µg/µl of DNA pCAG-GFP, 0.75 µg/µl Cdh11 or pCDNA3.0) in H_2_O containing 0.0125% fast green was injected through the uterine wall into the lateral ventricle of the embryos using a glass micropipette made from a microcapillary tube. After injection, Tweezer-trodes (BTX) were applied across the outside of the uterus–oriented to flank the embryonic brain, and five 50 ms square pulses of 30V with 950 ms intervals were delivered by an electroporator (BTX 830). Following injection and electroporation, the uterus was returned inside the abdomen and the abdominal muscle wall and skin sealed with sutures. Animals are allowed to survive 24 hours before analysis. Brains were fixed for 8–16 hr in 4% paraformaldehyde, cryoprotected in 30% sucrose dissolved in PBS for overnight and embedded in OCT. A cryostat was used to make 12 µm coronal sections.

### Glioma Stem Cell Isolation and Culture

Glioma stem cells (GSCs) were isolated from GBM primary surgical specimens and were mechanically and enzymatically dissociated, red blood cells were lysed using ACK buffer (Gibco), and a single cell suspension was achieved using a 100 µm strainer. Cells were plated adherently in laminin-coated flasks in NSC media (NS-A base media (Stem Cell Technologies), EGF and bFGF2 (Peprotech), B27 supplement (Invitrogen), and N2-A supplement (Stem Cell Technologies), according to methods detailed in [12].

### Production of Lentiviruses

TRC2 expression plasmids for lentivirus were acquired from Sigma.

shRNA sequence #1 against CDH11: CCGGTAAACTCTGGACACTCTATATCTCGAGATATAGAGTGTCCAGAGTTTATTTTTG (The RNAi Consortium sequence TRCN0000303363, clone NM_001797.2-3233s21c1).

shRNA sequence #2 against CDH11:

CCGGGACTTCATCAACACGAGAATACTCGAGTATTCTCGTGTTGATGAAGTCTTTTTG (The RNAi Consortium sequence TRCN0000303384, clone NM_001797.2-2589s21c1). Non-silencing shRNA (SHC202 MISSION® TRC2 Control Vector) contains a shRNA sequence that does not target human or mouse genes. Preceding plasmids contain a puromycin resistance cassette for selection. pGIPZ non-silencing shRNA (Open Biosystems #RHS4346) also expressed turboGFP. Lentiviral expression constructs were cotransfected with psPAX2 packaging plasmid and pMD2.G envelope plasmid into HEK293T cells, and packaged virus concentrated by centrifugation.

### Cell Culture

Glioma stem cells (GSCs) were isolated from GBM primary surgical specimens in keeping with protocols approved by the Northwestern University Institutional Review Board, NU 07C2: “Lineage determination of brain tumor stem-like cells (BTSCs) harvested from human astrocytic and oligodendroglial tumors.” Written informed consent was obtained from the donors or next of kin for the use of tissue samples for this research. The specimens were mechanically and enzymatically dissociated, red blood cells were lysed using ACK buffer (Gibco), and a single cell suspension was achieved using a 100 µm strainer. Cells were plated adherently in laminin-coated tissue culture flasks as detailed in [12]. Primary HUVEC and immortalized HUVECs (iHUVECs) were provided by the Muller lab (Northwestern Univ), mouse brain endothelial cells (mBends) from ATCC (bEnd.3 cells). For co-culture studies, GBM cells were infected with NSshRNA GIPZ lentivirus and sorted (Dako Cytomatiion MoFlo; Northwestern Robert H. Lurie Cancer Center Flow Cytometry Core) to purify GFP+ cells. 5×10^4^/cm^2^ GFP+ GBM cells were plated on a confluent monolayer of HUVEC, mBend, or GBM cells, and were cultured for 24 hours. For recombinant TGFβ1 studies, GBM cells were starved overnight from growth factors bFGF2 and EGF, then treated for 24 hours with recombinant Transforming Growth Factor (TGF)β1 (R&D Systems).

### Transwell Migration Assay

GBM cells were infected with NSshRNA or CDH11 lentivirus as described above and were selected with 1 ug/ml puromycin until cells achieved adequate knockdown of Cadherin11 as determined by western blot. To facilitate visual discrimination between NSshRNA and CDH11 shRNA lines, NSshRNA lines were colabeled with EFα-GFP lentivirus (Kessler lab, Northwestern University) and CDH11 shRNA lines were colabeled with BOB-mCherry lentivirus (Addgene). Fluorescently labeled cells were sorted using Fluorescence-activated cell sorting (FACS), and GFP+ or mCherry+ sorted cells were used for migration experiments. Cells were grown in media without growth factors for 24 hours prior to migration experiment. 2×10^4^ each of NSshRNA and CDH11 shRNA cells are plated in the top chamber of a Fluorblok migration insert (BD 351152) in media without growth factors. Serum free conditioned media from 3T3 fibroblasts was used in the bottom chamber as a chemoattractant. After 20 hours, cells are fixed in 4% PFA, and migrated cells are those cells on bottom side of the membrane. Recombinant Transforming Growth Factor (TGF)β1 was used at 5 ng/ml. After antibody staining, the membranes imaged on a Zeiss Confocal LSM510 microscope, with 13 fields per membrane imaged and counted for GBM77 lines, and 20 fields per membrane for GBM83 lines.

### Immunofluorescence and Immunohistochemistry

Brain sections were incubated with blocking solution (5% goat serum and 0.3% Triton X-100 in PBS) for 1 hour at room temperature, and then incubated with primary antibodies diluted in blocking solution for 2 hours at room temperature (for Pax6, Tbr2, Tbr1) or overnight at 4°C (GFP). Primary antibodies used were anti-CDH11 mouse monoclonal antibody (1∶100, Invitrogen 32–1700) anti-GFP chicken polyclonal antibody (1∶1000, Abcam, ab13970), anti-Pax6 mouse monoclonal antibody (1∶200, Developmental Studies Hybridoma), anti-Tbr2 rabbit polyclonal antibody (1∶250, Abcam ab23345), anti-Tbr1 rabbit polyclonal antibody (1∶200, Chemicon ab9616). After washing in PBS, the sections were then incubated with secondary antibodies and DAPI diluted in PBS for 1 hour at room temperature. Secondary antibodies used were Alexa488-conjugated goat anti-chicken IgG antibody (1∶1000, Molecular Probes), Alexa555-conjugated goat anti-mouse IgG antibody (1∶1000; Molecular Probes), Alexa555-conjugated goat anti-rabbit IgG antibody (1∶1000; Molecular Probes), Alexa647-conjugated goat anti-mouse IgG antibody (1∶1000; Molecular Probes), Alexa647-conjugated goat anti-rabbit IgG antibody (1∶1000; Molecular Probes), and Alexa488-conjugated goat anti-mouse IgG antibody (1∶1000; Molecular Probes). The analysis throughout the paper was carried out in a semi-blinded manner as previously described [13]. On each image, GFP+ electroporated cells were marked by green color with only the channels for GFP (green) and DAPI (blue) on. The position of these marked cells was then transferred to an image containing only the marker of interest (Tbr1, Tbr2 or Pax6) (the image contains red channel only and therefore shows monochrome), and the colocalization of these markers was noted. Cells are identified by colocalization of the fluorescent construct with DAPI (nuclear stain). Paraffin-embedded glioma samples from the Northwestern Memorial Hopsital Tissue Core were sectioned and rehydrated. After blocking, sections were incubated with mouse anti-Cadherin11 antibody (1∶100 Invitrogen 32–1700) overnight at 4°C, washed, and incubated with biotin-conjugated secondary antibody, and developed using DAB peroxidase kit (Vector). Tissue was counterstained with Heamatoxylin.

## Results

### CDH11 Induces Cell Migration from the Developing Cortical VZ *in vivo*

As differential expression of cell adhesion molecules has been implicated in the regulation of both normal and metastatic cell migration, we sought to identify changes in the expression of cell adhesion molecules as cells exit the VZ. To this end, we analyzed the expression and network interactions of genes differentially-regulated as cells exit the VZ, previously identified using fluorescence activated cell sorting (FACS), from GFP-expressing cells from embryonic E14.5 Eomes:: GFP transgenic mice (**Fig. 1C, D**) [**11**]. mRNA profiling from E14.5 GFP-expressing and GFP-negative cells from E13.5 cortex identified 21 genes for known adhesion molecules upregulated >3 fold, and 14 genes downregulated >3 fold [11].

As our previous studies in cortical development indicated that β-catenin signaling plays a critical role in the regulation of neural progenitor proliferation vs. differentiation [14], [15], we hypothesized that β-catenin signaling might function in a regulatory network that controls neuronal migration through the regulation of cell adhesion molecules. To test this hypothesis, we used a network analysis tool (IPA) to explore possible regulatory interactions between β-catenin and these dynamically-regulated adhesion molecules (**Fig. 1E** **).** In addition to β-catenin, all of the adhesion molecules from the > = 3-fold up- and down-regulated populations were imported into the pathway analysis tool and the network of direct and indirect interactions was identified. This analysis revealed adhesion molecules and signaling pathways with previously observed roles in neuronal migration and differentiation (e.g. Integrin signaling, ERK/MEK, TGFβ). We found that β-catenin was highly interconnected within this network of adhesion molecules, with direct protein-protein interactions with 10 of the set of regulated adhesion proteins. Of these genes, a mesenchymal cadherin, cadherin-11 (*Cdh11*) was upregulated 3.8-fold. CDH11 is a type-II classic cadherin that mediates homophilic cell-cell adhesion and has been implicated in EMT processes [16]. The expression of *Cdh11* in developing cortex was examined in E14.5 cortex, and CDH11 immunoreactivity was enriched outside the VZ in the subventricular (SVZ) and intermediate zones (IZ) where migrating multipolar neurons reside (**Figs. 1F and S** **1**). Together, the network analysis and expression patterns suggested that Cdh11 might play an important role in neuronal migration or exit from the VZ.

**Figure 2:**
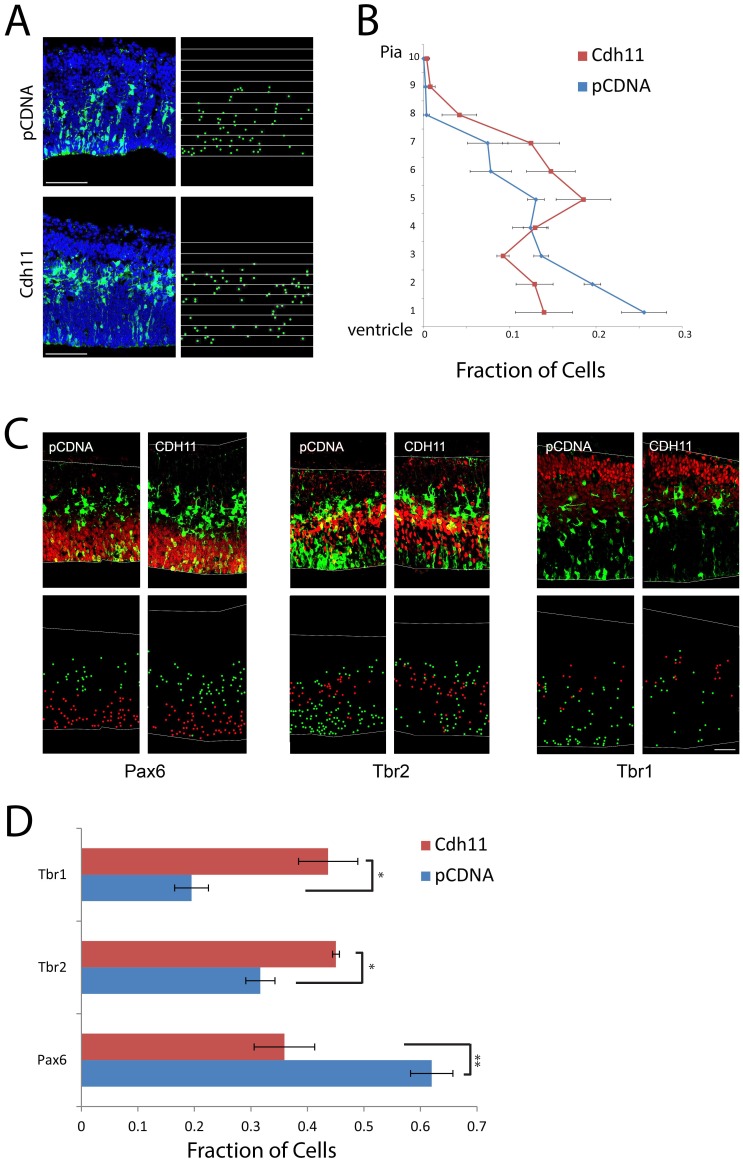
Overexpression of CDH11 in cortical VZ precursors causes premature exit from the VZ and neuronal differentiation. (**A**) E13.5 cortical precursors were electroporated in utero using either pCDNA control (N = 4) (top) or with pCAG-Cdh11 expression plasmid along with pCAG-EGFP plasmid (N = 4) and analyzed at E14.5. Electroporated cells were identified with antibody staining against GFP and sections counterstained with the DNA dye DAPI (pseudocolored blue). To quantify changes in cortical positioning of electroporated cells, ten equal sized bins were drawn over each image. Each white dot corresponds with the soma of an electroporated cell. Bar = 100 µm. (**B**) The fraction of the total GFP+ cells in each of the ten bins was then graphed for the two experimental conditions. Brackets indicate 1 SEM. N = 4 brains (PCDNA), 3 brains (Cdh11). (**C**) Sections were stained for radial glial marker Pax6, intermediate progenitor marker Tbr2, and neuronal marker Tbr1. Electroporated cells are pseudocolored green, and the respective antigens, red. Bar = 50 µm. The dot plots highlight the cell bodies of electroporated cells, with red representing electroporated cells that express the marker of interest and green indicating electroporated cells that do not express the marker. (**D**) Histograms represent fraction of total electroporated cells found in each brain region, showing the fraction of cells that express each marker after electroporation (red/(red+green)), showing premature neuronal differentiation. For Pax 6, Cdh11 vs. control (N = 4 brains for each, **P = 0.0072), Tbr2 (N = 3 for Cdh11, N = 4 for pcDNA control, *P = 0.077), and Tbr1 (N = 4 for each, *P = 0.0071). Unpaired T-test.

To test the role of CDH11 in the initiation of cell migration from the cortical VZ, we used *in utero* electroporation to overexpress CDH11 in cortical VZ cells. E13.5 embryos were co-electroporated with GFP and an expression plasmid driving *Cdh11* or pcDNA control and analyzed after 24 hours. Compared with control cells, CDH11-overexpressing cells prematurely exited the VZ (**Fig. 2A, 2B** **, S2, S3**) and underwent premature neuronal differentiation, as revealed by the altered expression of Pax6, Tbr2, and Tbr1 transcription factors (**Fig. 2C, 2C** **, S4–9**). Together, the observations that *Cdh11* is upregulated in cells exiting the VZ, and that CDH11 overexpression causes premature VZ exit and neuronal differentiation support that increased CDH11 can induce cell migration from the neuroepithelium during cortical development.

### CDH11 Overexpression in GBM Predicts Reduced Survival

**Figure 3:**
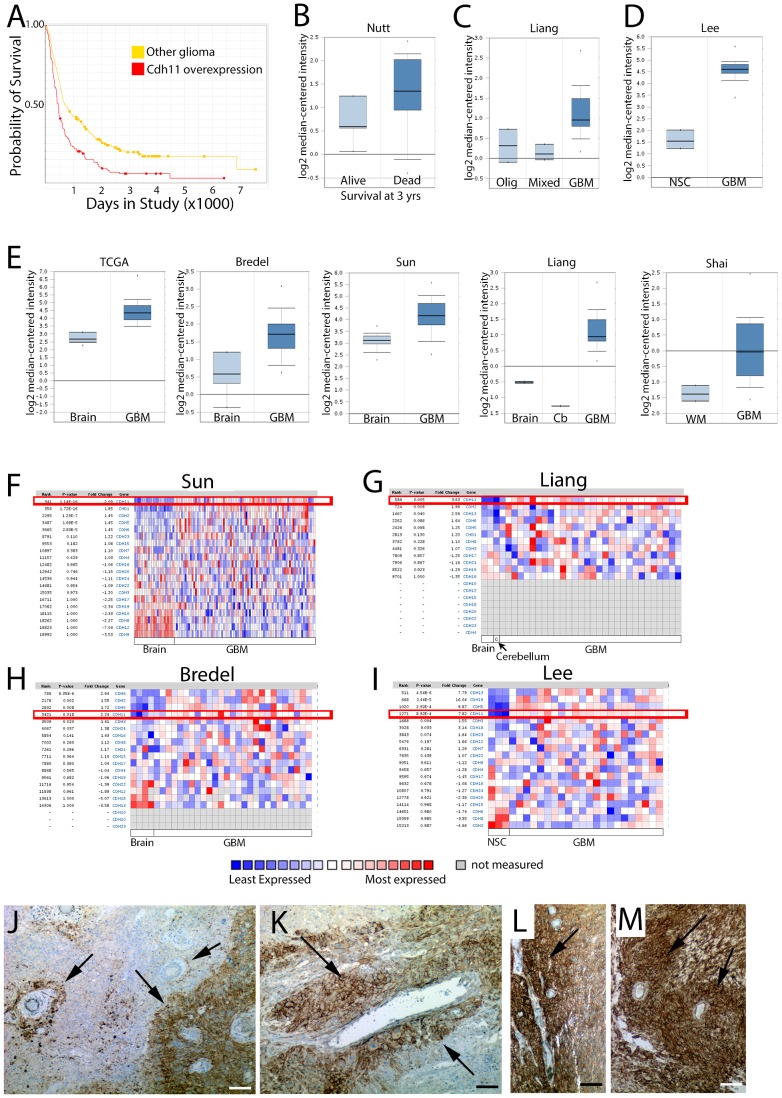
CDH11 is differentially expressed in human glioma vs. normal brain, and overexpression confers worsened prognosis. (**A**) Kaplan-Meier Survival Plot for samples with differential *CDH11* gene expression reveal that glioma patients with overexpression (red curve) have a worse prognosis than patients with intermediate expression. Log-rank P-value for Up-Regulated vs. Intermediate = 3.8535E-6. Data from NCI 2005<http://rembrandt.nci.nih.gov>. (**B**) *CDH11* expression is predictive for survival in GBM; fold-change 3.6, P = 0.005 by T-test (Data from [38]). (**C**) *CDH11* expression is higher in GBM vs. mixed glioma or oligodendroglial tumors; fold-change = 1.8, P = 5.83×10^−4^ by T-Test (Data from [39]). (**D**) *CDH11* expression is higher in GBM vs. neural stem cells. Fold change = 7.8, P = 8.92×10^−4^ (Data from [40]). (**E**) *CDH11* is relatively overexpressed in GBM vs. normal brain tissue. TCGA glioblastoma dataset analysis shows 2.9 fold overexpression of CDH11 (P = 1.14×10^−10,^ T-Test). GBM vs. Normal in (Bredel [41]): 2.2 fold increase; P = 0.018 (T-test). GBM vs. Normal (Sun [42]): 2.1 fold increase (P = 1.14×10^−16^, T-test). GBM vs. normal (Liang [39]): 3.6 fold increase (P = 0.005, T-test). GBM vs. WM (Shai [43]): 2.5 fold overexpression compared to white matter (P = 9.75×10^−8^; T-test). (**F–I**) Relative expression of cadherin genes in GBM vs. normal from [42] (**F**), [39] (**G**), [41] (**H**), and [40] (**I**) reveals that CDH11 (boxed in red) is differentially regulated in GBM vs. normal across multiple datasets. (**J–M**) Immunoperoxidase staining for CDH11 expression in human GBM tissue show heterogenous expression patterns, with enrichment of staining in tumor cells adjacent to tumor vessels (arrows). Cells surrounding vessels of varying sizes from small (**J**) to large vessels (**K**) express Cdh11. As typical for GBM, the tumor histoarchitecture is highly varied, with tissue showing variable necrosis and marked heterogeneity of cellularity (notably (**J**) and (**K**)). Bar = 100 µm.

As inhibiting the extensive cellular invasion in GBM presents a clinically significant therapeutic target, we sought a greater understanding of the underlying mechanisms that stimulate GBM cell motility. As a number of studies have suggested that GBM cells resemble cortical progenitor cells, and may share regulation pathways [17]–[19], we hypothesized that molecules such as CDH11 that regulate cell migration in the developing cortex might play an important role in GBM. We analyzed survival data from tumor gene expression databases (NCI Rembrandt, Oncomine) and found that increased *CDH11* expression predicts reduced survival in glioma patients (**Fig. 3A–B**). We also found that GBMs have higher expression of *CDH11* than mixed gliomas and oligodendroglial tumors (**Fig. 3C** **)**. Furthermore, GBMs have higher expression of *CDH11* than normal whole brain tissue, cerebellar tissue, white matter, and neural stem cells (**Fig. 3D–E** **).** Comparison of the relative expression of cadherins revealed that *CDH11* is consistently one of several cadherins overexpressed in GBM vs. normal brain (**Fig. 3F–H** **, S10**) or GBM vs. NSC (**Fig. 3I** **and S10**). Together, these expression data are consistent with the idea that *CDH11* expression levels are increased in brain tumors and correlate with brain tumor grade and suggest a function for CDH11 in brain tumor aggressiveness.

### Endothelial Cells Stimulate CDH11 Expression and TGFβ Signaling in GBM Cells

To examine the cellular expression of CDH11 in human glioblastoma, we performed immunoperoxidase staining for CDH11 on human tumor samples. These studies revealed heterogeneous expression patterns in human GBM specimens. Within individual tumors, CDH11 immunoreactivity ranged from absent to densely staining (**Fig. 3J–K**), with high variability of both the intensity and fraction of cells staining positive. In many areas, intense Cdh11 immunoreactivity was observed in tumor cells in adjacent to tumor vessels. Increased microvessel density correlates with poor patient survival in astroglial brain tumors [20], and recent data has suggested that vascular endothelial cells provide critical cues to promote GBM aggressiveness [21]. Together, these observations suggested the possibility that endothelial cells may stimulate CDH11 expression.

**Figure 4:**
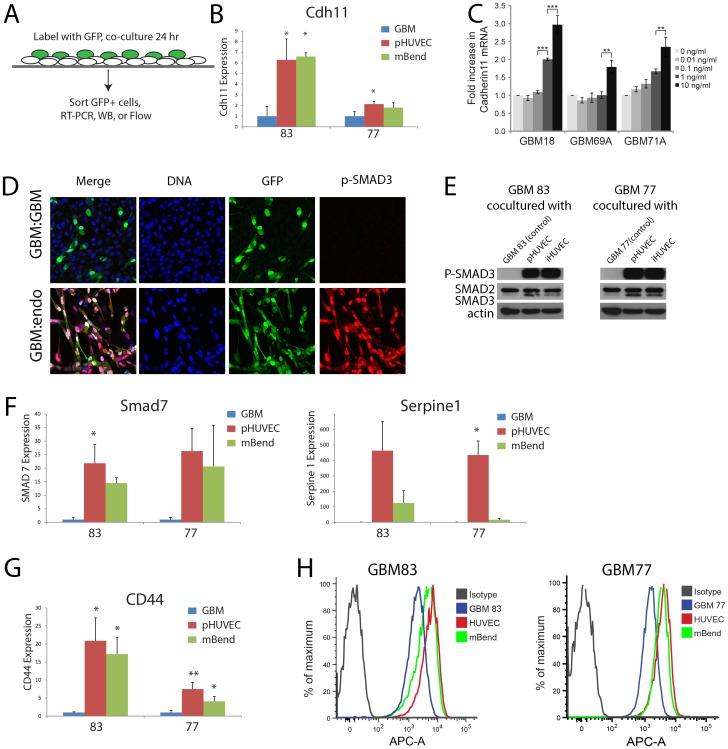
CDH11 expression is upregulated by endothelial cells and TGFβ. (**A**) Primary human GBM cells are labeled with GFP and co-cultured with either unlabeled GBM cells or endothelial cells. GFP expressing cells are then purified by fluorescence activated cell sorting and CDH11 expression is measured by qRT-PCR. (**B**) Coculture with endothelial cells (HUVEC, mBend) causes increased expression of CDH11 in primary human GBM cells (GBM line 83 P = 0.0164 by ANOVA (n = 5); Line 77, P = 0.0284 by ANOVA (n = 6)). (**C**) *CDH11* mRNA is increased in a dose-dependent fashion by TGFβ. (N = 3 for each cell line. Line 18 P<0.0001; Line 69A P = 0.0001; Line 71A P = 0.0005; all by Repeated Measures ANOVA). (**D**) Endothelial cell co-culture induces p-SMAD3 expression in GBM cells (top row: GBM co-cultured upon GBM; bottom row: GBM cultured with endothelial cells). Bar = 50 µm. (**E**) Western blots confirm induction of SMAD3 phosphorylation by co-culture of GBM with endothelial cells. (**F**) TGFβ transcriptional targets SMAD7 (Line 83, P = 0.0275 by ANOVA (n = 4); Line 77 n = 4) and Serpine-1 (Line 77, P = 0.0082 by Repeated Measures ANOVA (n = 3); Line 83 n = 4) are upregulated in GBM co-cultured with endothelial cells (primary HUVEC or mouse brain endothelial cell line mBend). (**G**) CD44 mRNA (Line 83 P = 0.0196 by ANOVA (n = 5); line 77 P = 0.0017 by ANOVA (n = 4)) and (**H**) cell surface expression (measured by flow cytometry) is upregulated after endothelial co-culture. For all pairwise comparisons, Newman Keuls posthoc tests were used, * P<0.05, ** P<0.01, *** P<0.001 and refer to comparison with control (GBM-GBM) unless otherwise noted.

To test if endothelial cells can regulate CDH11 levels in GBM cells, we co-cultured primary GBM cells directly with endothelial cells. GBM cells were labeled with GFP so that the GBM cells could be isolated by FACS using GFP fluorescence after co-culture. We observed that after 24 hrs co-culture, GBM cells incubated with human umbilical vein endothelial cells (HUVECs) or mouse brain endothelial cells (mBend) showed increased *CDH11* expression compared with coculture with GBM cells alone (**Fig. 4A, B**).

The TGFβ signaling pathway is an attractive candidate signaling pathway that may regulate CDH11 expression by endothelial cells. TGFβ signaling plays a critical role in both normal and pathogenic angiogenesis [22], and TGFβ activity confers a poor prognosis in GBM [23], [24]. We found a dose-dependent upregulation of *CDH11* mRNA expression to recombinant TGFβ in primary GBM cells (**Fig. 4C**).

To test if endothelial cells could induce TGFβ signaling in GBM cells, we examined markers of TGFβ signaling in GBM cells after co-culture with endothelial cells. Endothelial cells induced SMAD3 phosphorylation in GBM cells, providing support that endothelial cells can stimulate TGFβ signaling in GBM cells **(** **Fig. 4D–E** **)**. Real-time RT-PCR confirmed that endothelial cells also stimulated expression in co-cultured GBM cells of other TGFβ transcriptional targets SMAD7 and Serpine1 (also known as Plasminogen-Activator-Inhibitor-1) in the primary GBM cells (**Fig. 4F**). Another downstream target of TGFβ, CD44, functions in glioma cell migration and invasion [25], [26]. Here, we found that endothelial cell co-culture also stimulated the expression of *CD44* (**Fig. 4G**) and resulted in increased surface expression of CD44 protein (**Fig. 4H**). Together, these findings suggest that endothelial cells can function as a source of TGFβ to stimulate CDH11 in GBM cells.

### CDH11 Regulates TGFβ-induced GBM Cell Motility

**Figure 5:**
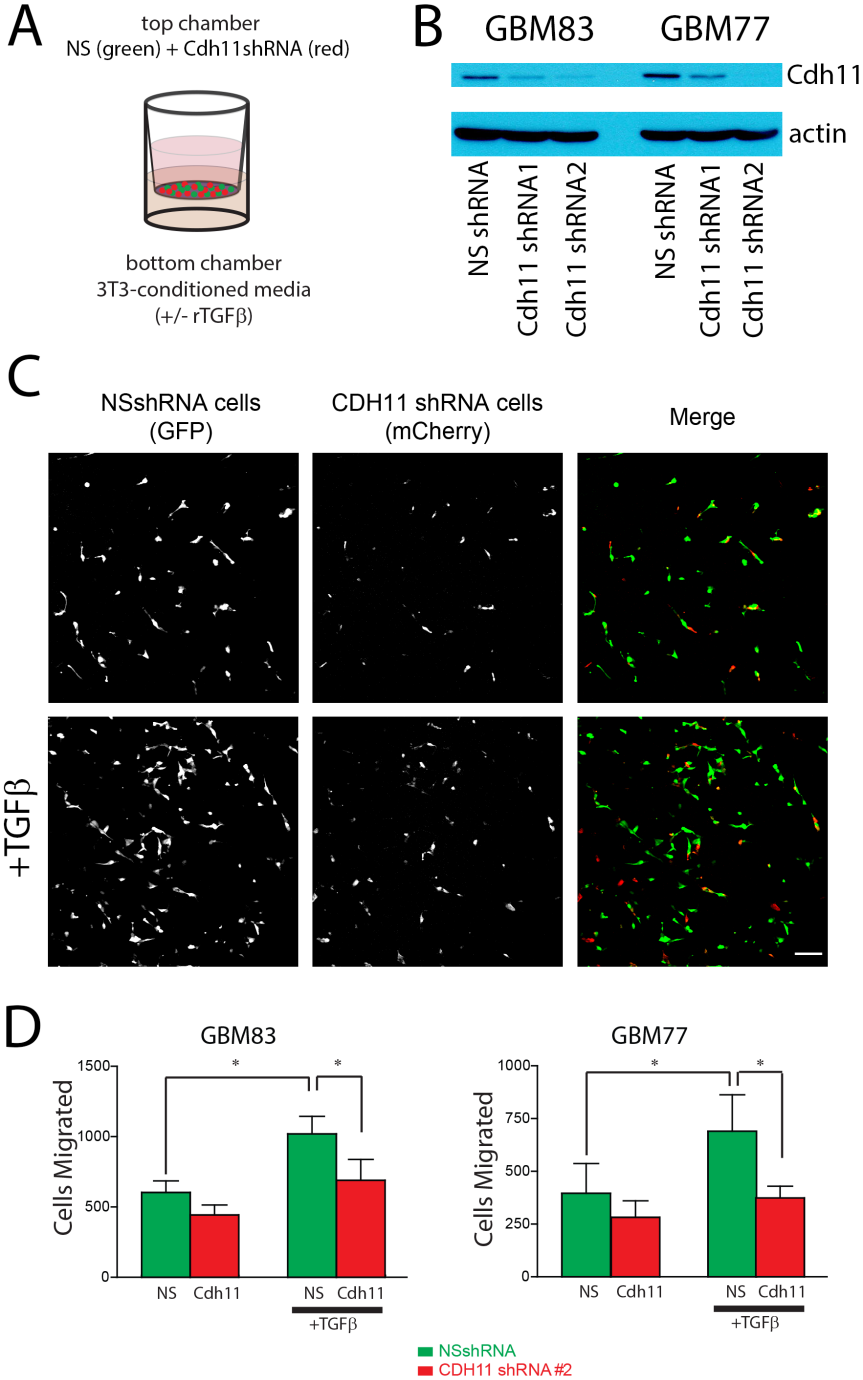
CDH11 knockdown reduces TGFβ-induced glioma cell motility. (**A**) Equal numbers of GBM cells expressing non-silencing shRNA and mCherry are seeded on the top of a porous polycarbonate membrane with GBM cells expressing shRNA to CDH11 and GFP and allowed to migrate towards bottom chamber with 3T3-conditioned. (**B**) Western blot confirming protein knockdown in shRNA treatments. (**C**) Images from the bottom of the polycarbonate filter showing control NS cells and CDH11 knockdown cells, quantified in (**D**) N = 3 biological replicates for each cell line; repeated measures ANOVA P = 0.01 (Line 83); 0.0308 (Line 77); * P<0.05 (Newman-Keuls posthoc test).

Our experiments showing that CDH11 overexpression stimulated migration of cortical cells from the VZ suggested that CDH11 could regulate motility of GBM cells. To test the role of CDH11 in GBM cell motility, we used shRNA lentiviruses to knock down CDH11 expression in primary GBM cells and assessed their motility using Transwell migration assays. Control non-silencing shRNA expressing cells labeled with GFP and CDH11 shRNA cells labeled with mCherry lentiviruses were plated in equal numbers of cells in transwell chambers **(** **Fig. 5** **)**. As the role of TGF-β in tumor promotion is associated with its function in inducing EMT-like increases in tumor cell motility [1], we tested the motility of GBM cells with and without TGF-β addition (**Fig. 5C, D**). We found that TGF-β increased the motility of GBM cells, and that CDH11 knockdown inhibits TGF-β-stimulated cell motility.

## Discussion

### EMT in Cortical Development and GBM

Epithelial to mesenchymal transitions have been described to drive the formation of many developing tissues and organs. Whether EMT changes in all tissues are regulated by conserved molecular mechanisms remains poorly understood. Although differentiating cortical cells undergo morphologic changes suggestive of EMT occurring in other developing organs, specific molecular differences distinguish the cortical neuroepithelial-mesenchymal transitions (“NMT”) from “classical” EMT. While a common feature of all EMTs (including NMT) is the loss of cadherin-containing adherens junctions [1], the predominant cadherin expressed in the cortical VZ is N-cadherin, with little to no detectable E-cadherin [8]. Upregulation of N-cadherin has been associated with increased motility in EMT [27], consistent with a pro-migratory function for N-cadherin in EMT. However, in several neuronal systems, loss of N-cadherin leads to increased cell migration, suggesting that the developmental and tissue context also influence whether specific cadherin isoforms promote or inhibit migration [28]–[31]. In the developing cortex, shRNA or conditional deletion of N-cadherin [13] or αE-catenin [32] also lead to increased migration. Together, these studies provide evidence that N-cadherin-containing adherens junctions in neuroepithelia function similarly to E-cadherin-based junctions in other epithelia to maintain the epithelial (non-motile) state.

Our findings of CDH11 upregulation in cortical cells stimulating migration support previous studies showing that modulation of cadherin adhesion can drive cell motility [16]. In many EMT’s, downregulation of E-cadherin at adherens junctions is accompanied by upregulation of other “mesenchymal” cadherins, including N-cadherin and Cdh11 [1]. In cortical cells, instead of an overt downregulation of N-cadherin expression, destabilization of N-cadherin adhesion by p35-Cdk5 kinase can promote migration [33]. In addition to *Cdh11* upregulation, in cortical development, we observed previously a switch from αE-catenin to αN-catenin as cells exited and migrated from the VZ [9]. While the functional consequences of switching of α-catenin isoforms remain poorly understood, these findings suggest that further understanding of regulators of cadherin adhesion can provide additional insights onto the mechanisms initiating migration.

### Endothelial Cells and GBM

Although the normal cues regulating CDH11 in development remain to be explored, previous studies indicating that endothelial cells stimulate both cortical stem cell self-renewal and neurogenesis (neuronal differentiation) suggest a complex regulatory function for endothelial cells. Our findings expand on previous studies to propose that endothelial cells not only support the self-renewal of tumor stem cells [21], they also stimulate cell motility via CDH11. As increased motility and stem cell properties are characteristic changes in tumors stimulated to undergo EMT [2], our studies are consistent with a broader interpretation of tumor “stemness” and the role of the microenvironment in brain tumors.

Our findings support recent studies suggesting that mesenchymal transitions may represent a general biological mechanism present in the invasive stage of many solid cancers [34]. Although our data suggest that TGFβ from endothelial cells is one mediator of CDH11 upregulation, there are undoubtedly other factors and mechanisms that can stimulate mesenchymal changes. A recent study in GBM cell lines provided evidence that CDH11 is upregulated in response to a fundamental activator of EMT, the bHLH transcription factor Twist1 [35]. Our findings from normal developing cortical progenitors that progenitor-progenitor interactions can stimulate their self-renewal [13] support observations that glioma initiating cells maintain their own tumorigenicity via TGFβ signaling [36]. As CDH11 upregulation is likely only one of multiple mesenchymal characteristics, it would appear unlikely that CDH11 or any individual molecule will prove to be the sole determinant of tumor aggressiveness. In addition to CDH11, we found differences in the expression of multiple cadherin molecules in GBM vs. normal brain, suggesting that regulation of the overall cadherin cell-adhesion expression landscape may play a crucial role in tumor development.

## Supporting Information

Figure S1 Images merged for Fig. 1F. (PDF)

Figure S2 Images merged for Fig. 2A (pCDNA electroporation). (PDF)

Figure S3 Images merged for Fig. 2A (Cdh11 shRNA electroporation). (PDF)

Figure S4 Images merged for Fig. 2C (pCDNA electroporation, Pax6 staining). (PDF)

Figure S5 Images merged for Fig. 2C (Cdh11 shRNA electroporation, Pax6 staining). (PDF)

Figure S6 Images merged for Fig. 2C (pCDNA electroporation, Tbr2 staining). (PDF)

Figure S7 Images merged for Fig. 2C (Cdh11 shRNA electroporation, Tbr2 staining). (PDF)

Figure S8 Images merged for Fig. 2C (pCDNA electroporation, TBr1 staining). (PDF)

Figure S9 Images merged for Fig. 2C (Cdh11 shRNA electroporation, Tbr1 staining). (PDF)

Figure S10 Higher magnification images of data presented in Figure 3. (PDF)
