# Supplementary figures and images for: Cadherin-11 Regulates Motility in Normal Cortical Neural Precursors and Glioblastoma
Source: PLoS One. 2013 Aug 7;8(8):e70962. doi: 10.1371/journal.pone.0070962 (PMC3737231; doi:10.1371/journal.pone.0070962)

## Supporting Information

Fig. S1

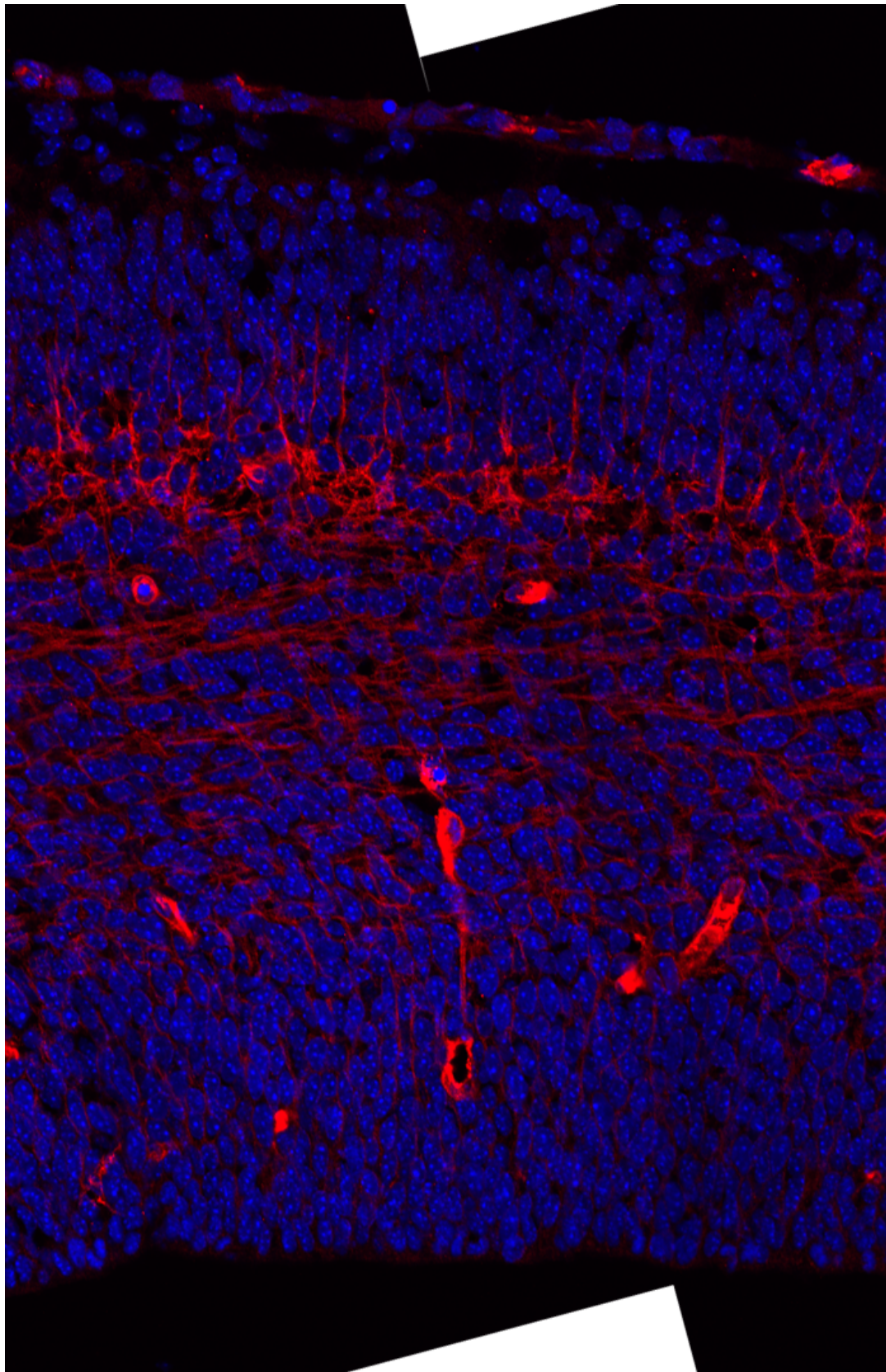

Supplement: Figure S1 — (PDF) [file pone.0070962.s001.pdf]

Figure S2.

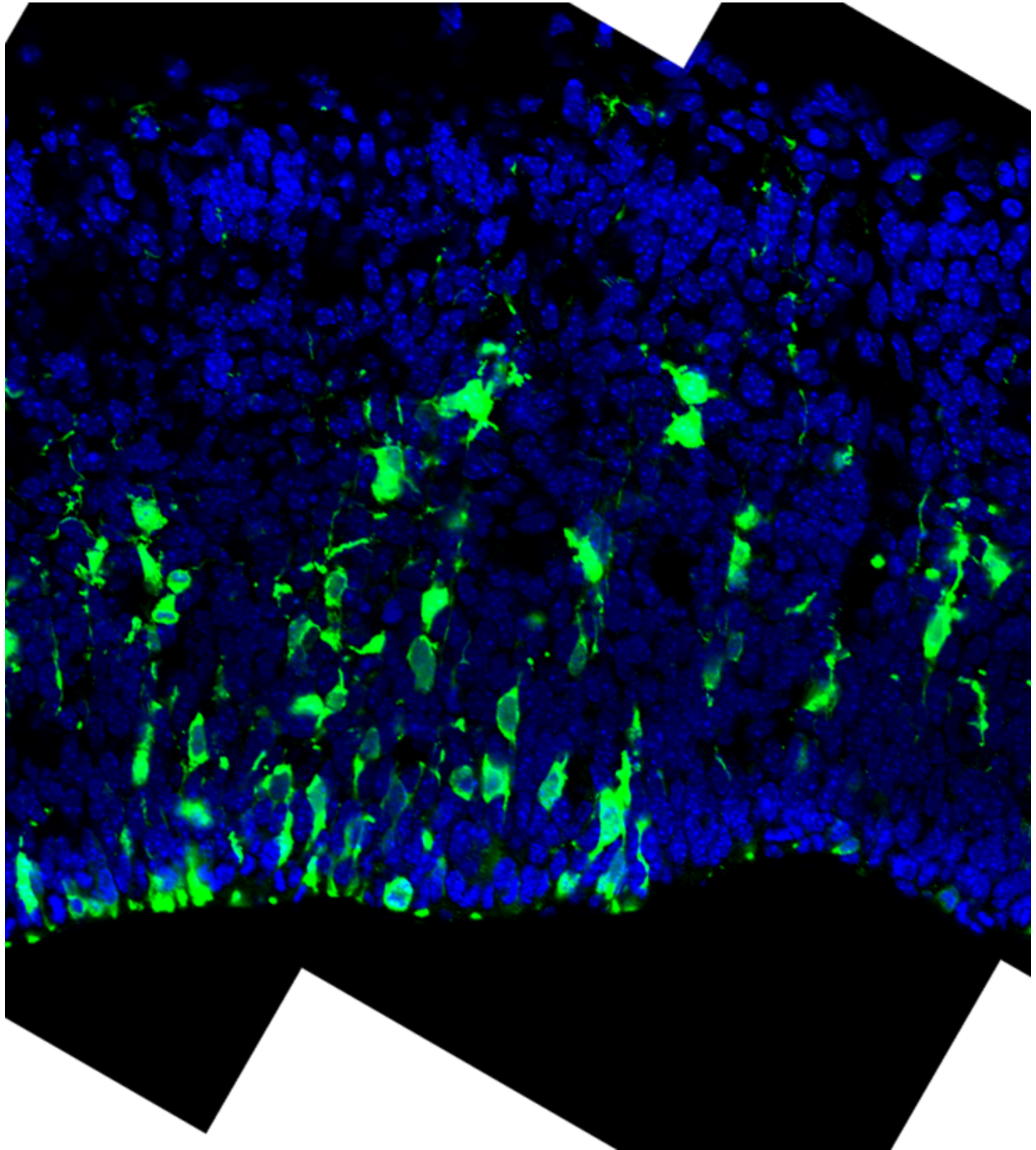

Supplement: Figure S2 — (PDF) [file pone.0070962.s002.pdf]

Fig. S3

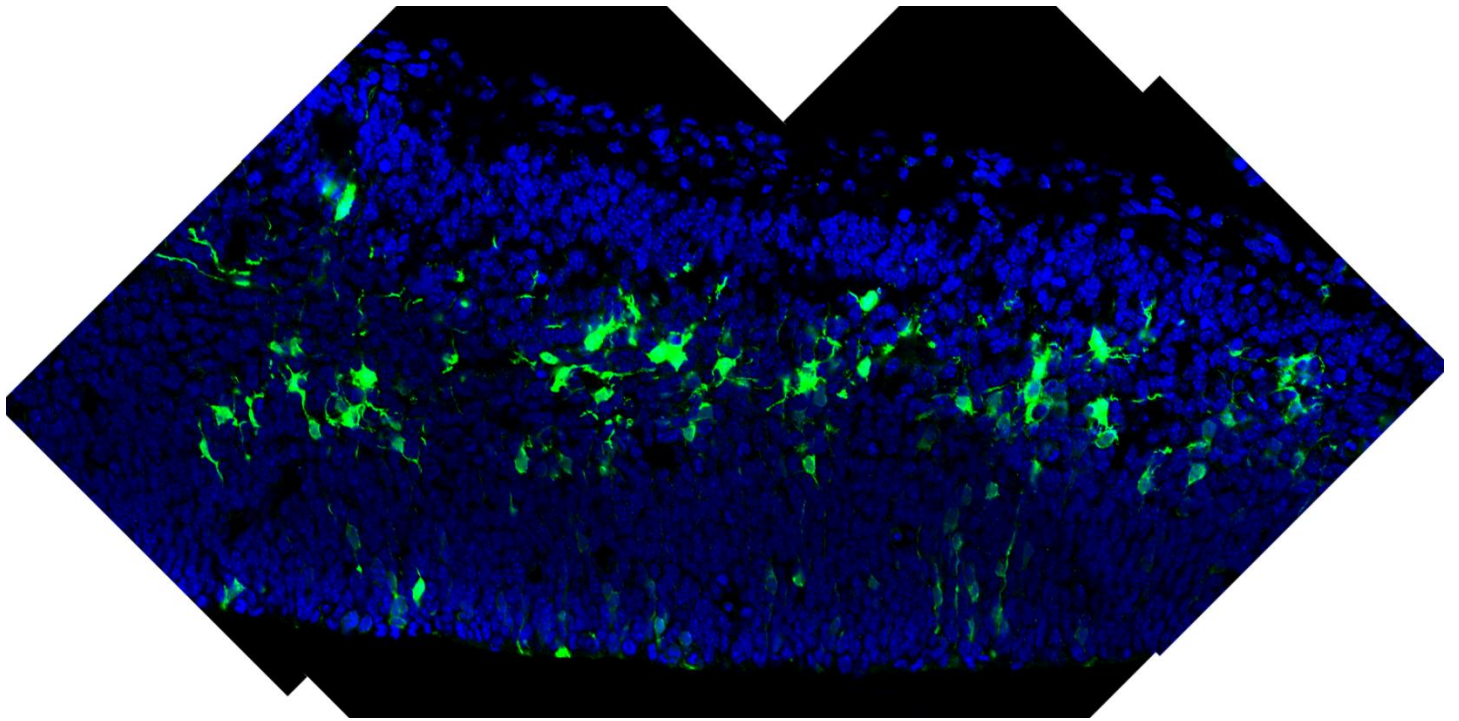

Supplement: Figure S3 — (PDF) [file pone.0070962.s003.pdf]

Fig. S4

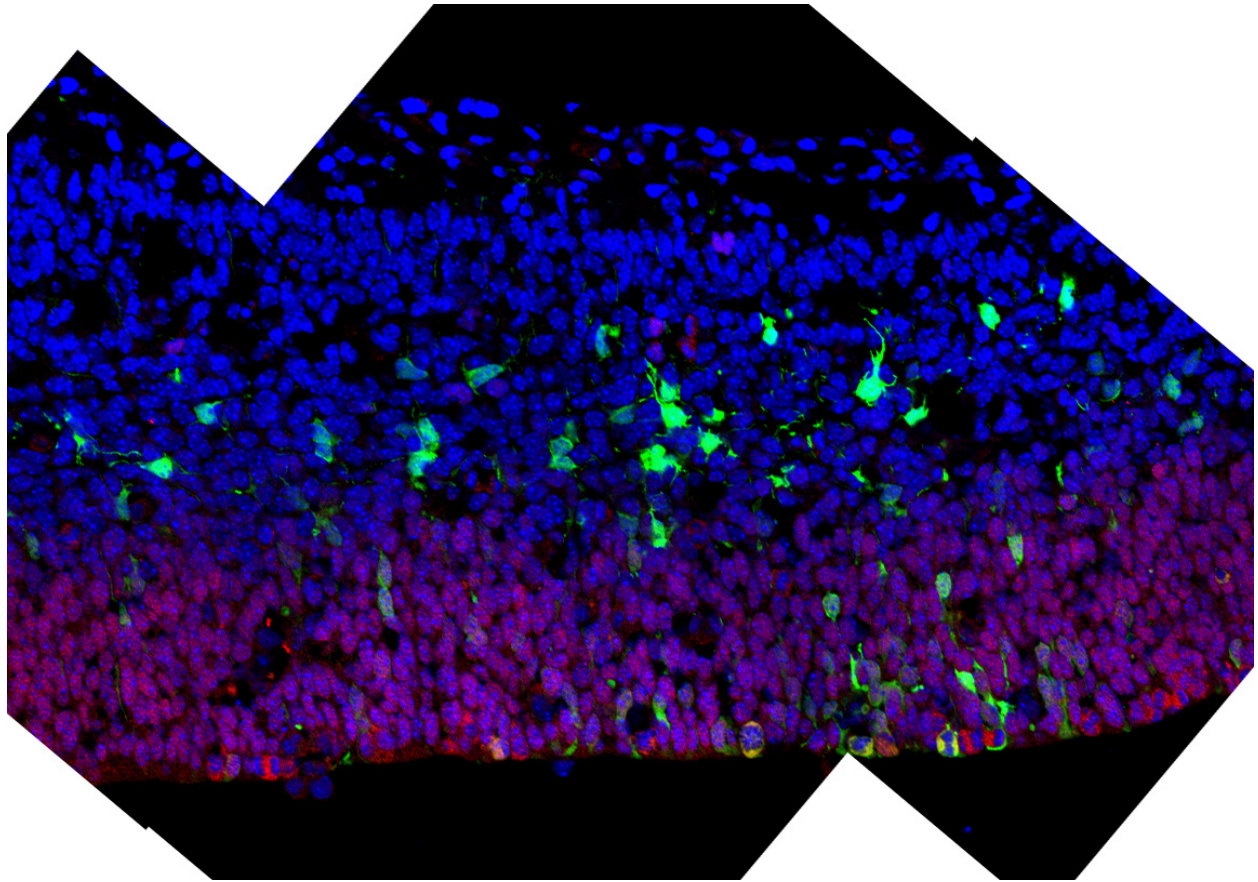

Supplement: Figure S4 — (PDF) [file pone.0070962.s004.pdf]

Fig. S5

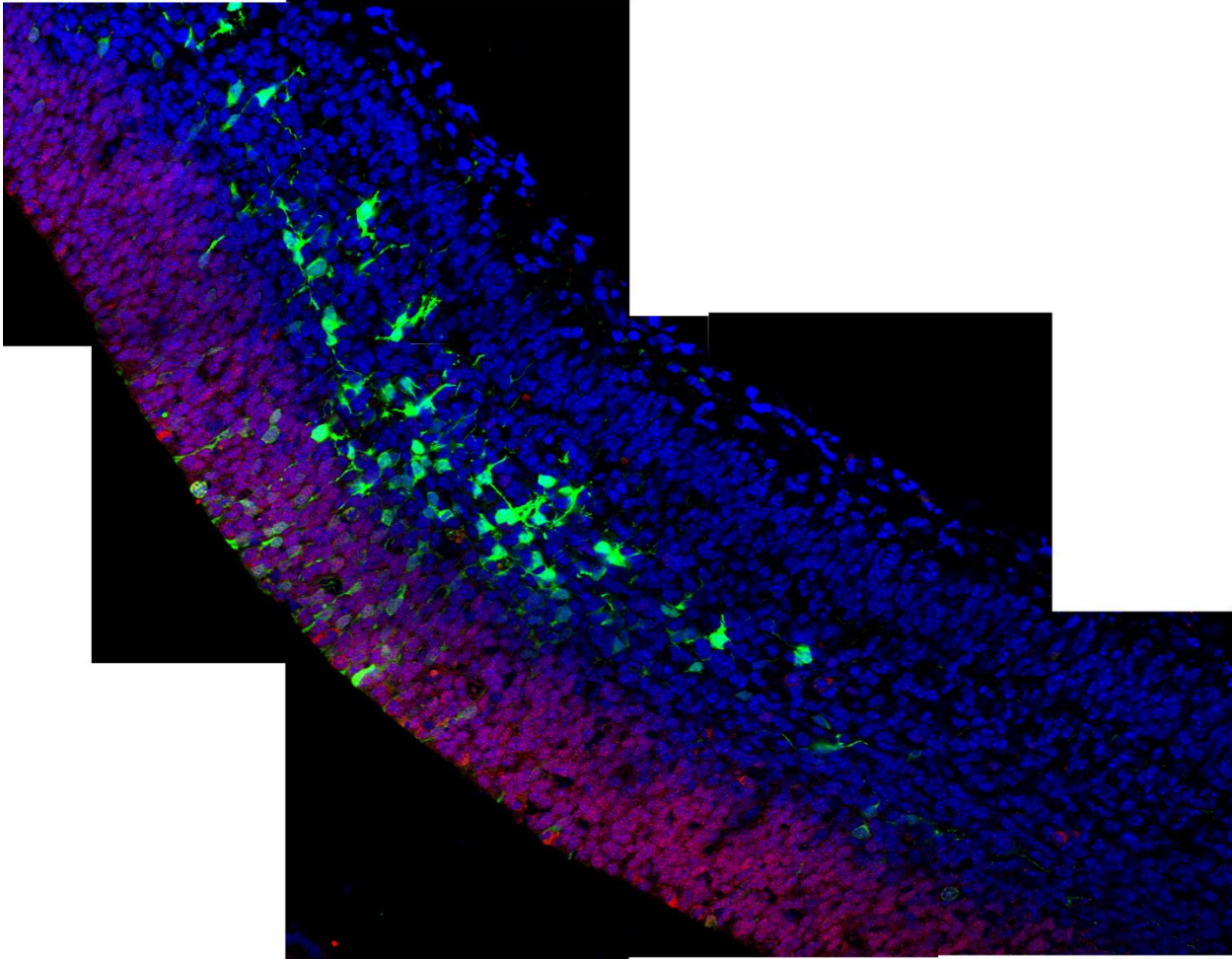

Supplement: Figure S5 — (PDF) [file pone.0070962.s005.pdf]

Fig. S6

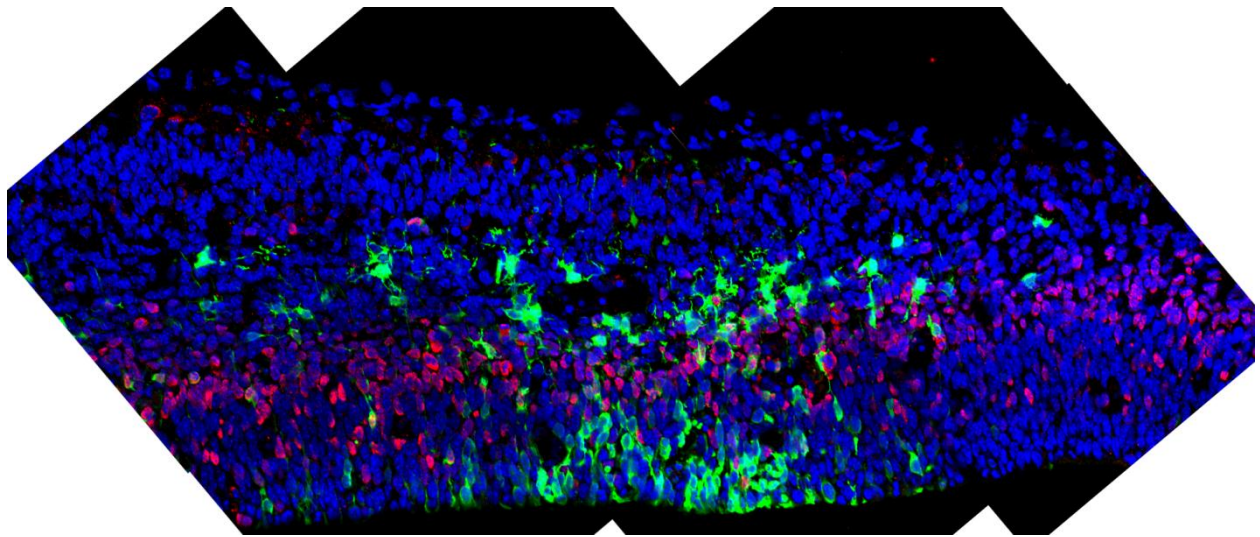

Supplement: Figure S6 — (PDF) [file pone.0070962.s006.pdf]

Fig. S7

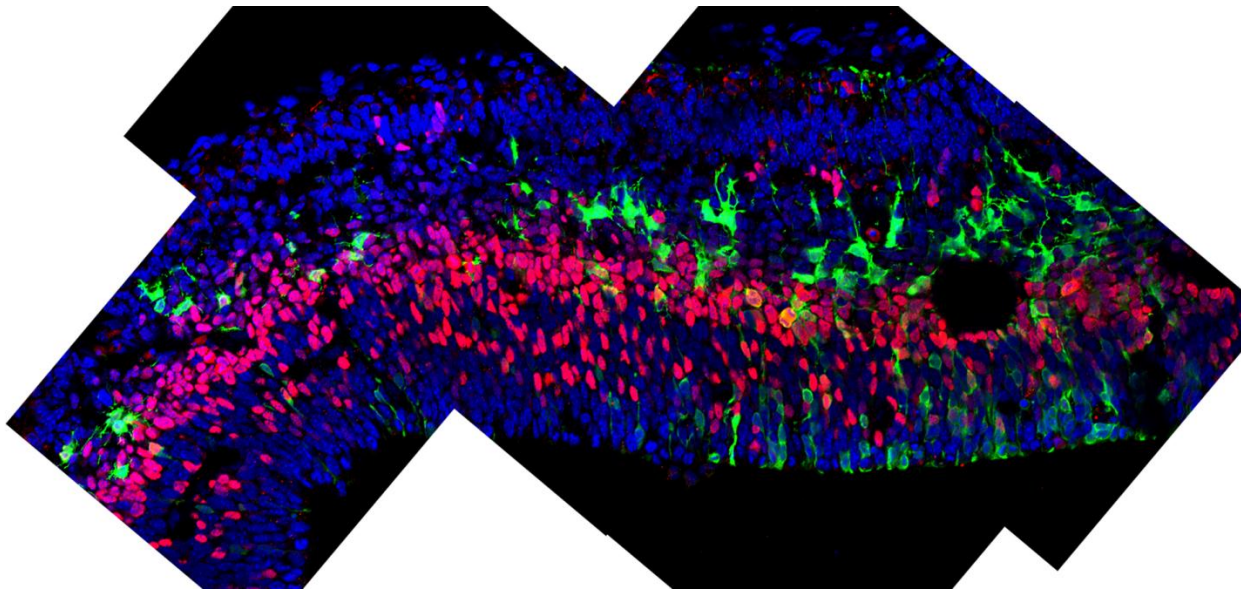

Supplement: Figure S7 — (PDF) [file pone.0070962.s007.pdf]

Fig. S8

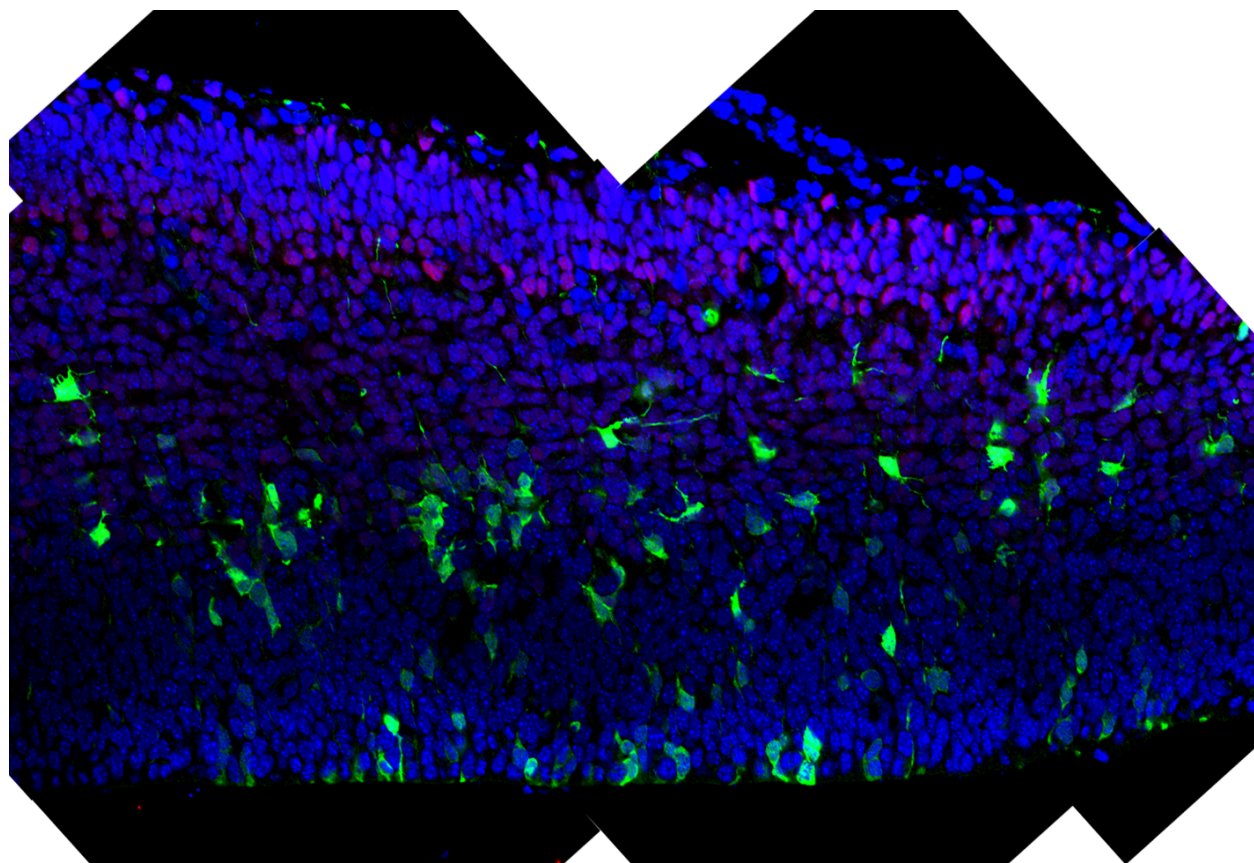

Supplement: Figure S8 — (PDF) [file pone.0070962.s008.pdf]

Fig. S9

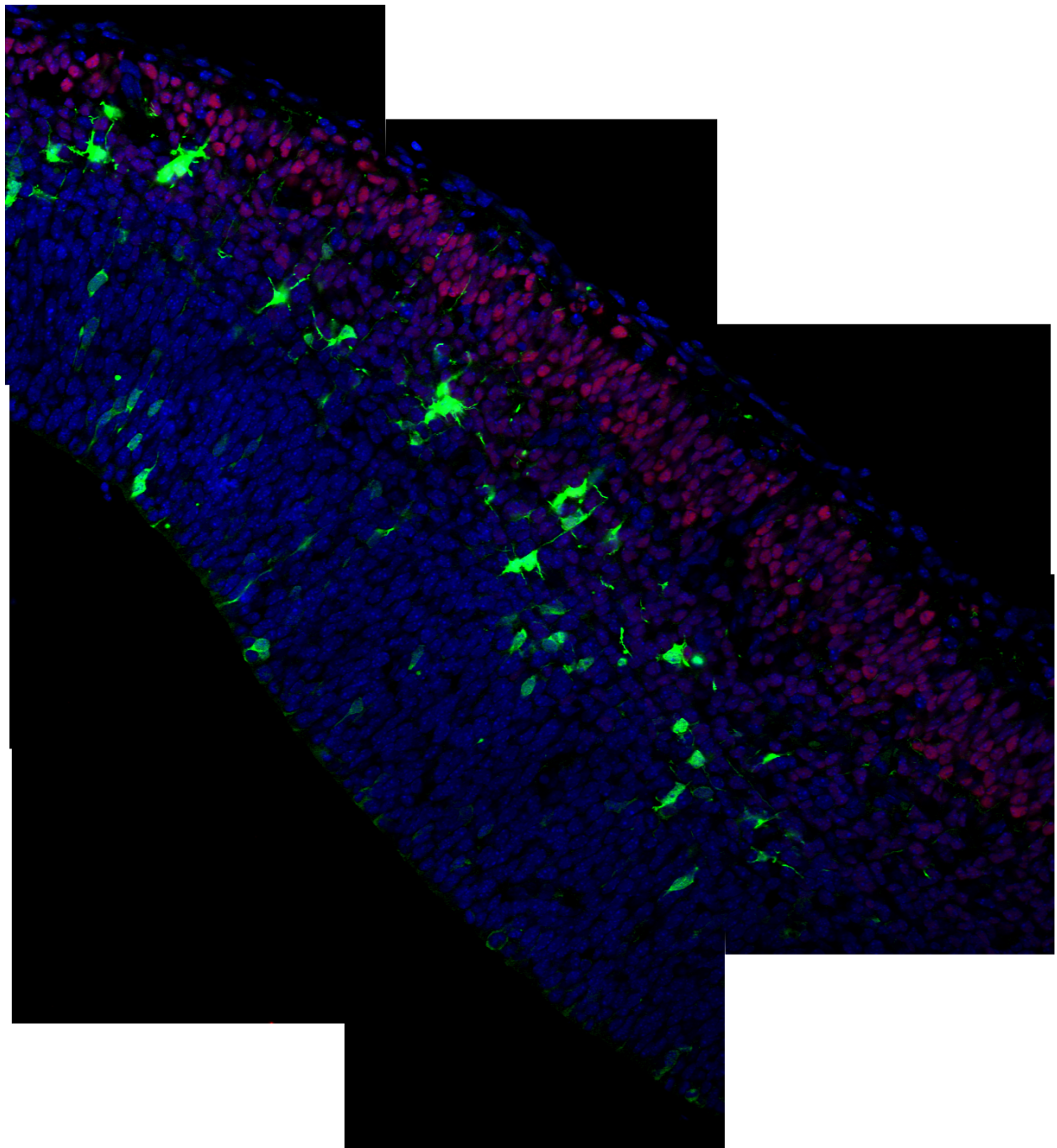

Supplement: Figure S9 — (PDF) [file pone.0070962.s009.pdf]
