# Supplementary material for: Cadherin-11 Regulates Motility in Normal Cortical Neural Precursors and Glioblastoma
Source: PLoS One. 2013 Aug 7;8(8):e70962. doi: 10.1371/journal.pone.0070962 (PMC3737231; doi:10.1371/journal.pone.0070962)
Supplement: Figure S10 — (PDF) [file pone.0070962.s010.pdf]

Fig. S10. Supplemental Data for Figure 3

Comparison of Selected Genes in Sun Brain

Over-expression in Glioblastoma vs. Normal

Rank	P-value	Fold Change	Gene	Reporter	Gene
341	1.14E-16	2.09	CDH11	207173_x_at	CDH11
356	1.72E-16	1.85	CHD1	204258_at	CHD1
2295	1.23E-7	1.45	CDH2	203440_at	CDH2
3487	1.69E-5	1.45	CDH5	204677_at	CDH5
3665	2.83E-5	1.45	CDH6	205532_s_at	CDH6
8791	0.110	1.22	CDH23	232845_at	CDH23
9553	0.182	1.08	CDH15	206328_at	CDH15
10897	0.383	1.10	CDH7	1554700_at	CDH7
11157	0.429	1.03	CDH4	239485_at	CDH4
12482	0.665	-1.06	CDH16	206517_at	CDH16
12942	0.746	-1.15	CDH20	210913_at	CDH20
14536	0.944	-1.11	CDH24	1553166_at	CDH24
14681	0.954	-1.09	CDH22	1569679_at	CDH22
15035	0.973	-1.20	CDH3	203256_at	CDH3
16711	1.000	-2.25	CDH17	209847_at	CDH17
17062	1.000	-2.34	CDH19	1555403_a_at	CDH19
18115	1.000	-2.33	CDH10	220115_s_at	CDH10
18262	1.000	-2.27	CDH8	210518_at	CDH8
18823	1.000	-7.04	CDH12	207149_at	CDH12
18992	1.000	-5.53	CDH9	207729_at	CDH9

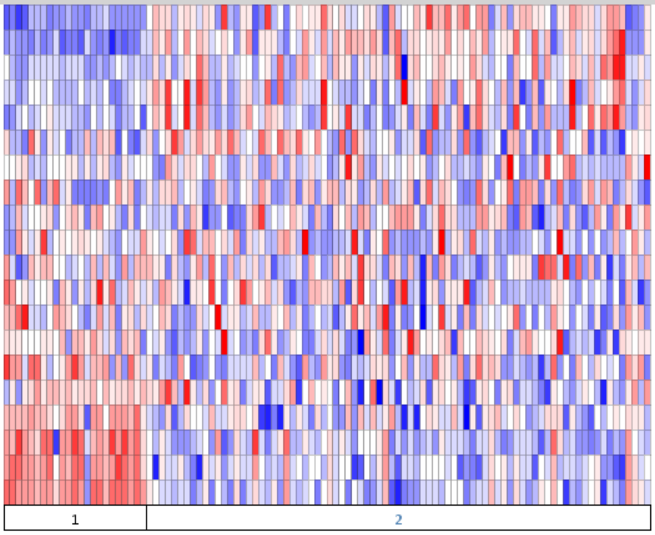

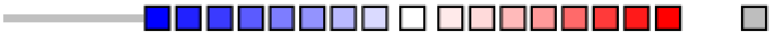

Note: Colors are z-score normalized to depict relative values within rows. They cannot be used to compare values between rows.

Legend

1. Brain (23) 2. Glioblastoma (81)

Cancer Cell 2006/04/01

mRNA

Human Genome U133 Plus 2.0 Array

180 samples

19,574 measured genes

Comparison of Selected Genes in Liang Brain

Over-expression in Glioblastoma vs. Normal

Rank	P-value	Fold Change	Gene	Reporter	Gene
586	0.005	3.63	CDH11	H96738	CDH11
724	0.009	1.96	CDH2	W48793	CDH2
1467	0.040	2.56	CDH13	AA160651	CDH13
2262	0.086	1.64	CDH6	AA421819	CDH6
2426	0.098	1.25	CDH5	T53625	CDH5
2813	0.130	1.20	CHD1	N56947	CHD1
3782	0.228	1.10	CDH8	R56219	CDH8
4481	0.326	1.07	CDH3	AA425217	CDH3
7809	0.857	-1.25	CDH17	AA088861	CDH17
7906	0.867	-1.16	CDH24	AI668564	CDH24
8522	0.923	-1.29	CDH19	N26740	CDH19
9701	1.000	-1.35	CDH16	AA919087	CDH16
-	-	-	CDH10	-	CDH10
-	-	-	CDH12	-	CDH12
-	-	-	CDH15	-	CDH15
-	-	-	CDH18	-	CDH18
-	-	-	CDH20	-	CDH20
-	-	-	CDH22	-	CDH22
-	-	-	CDH23	-	CDH23
-	-	-	CDH4	-	CDH4

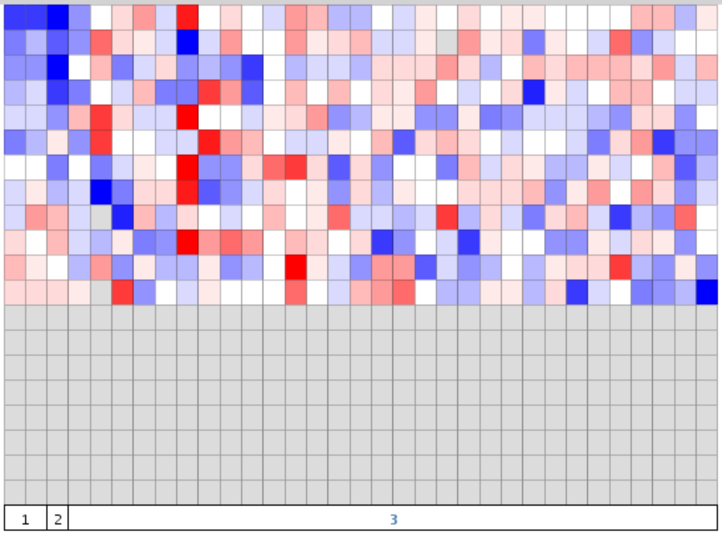

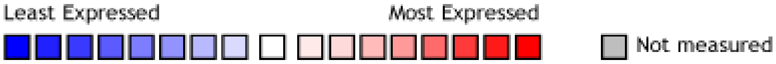

Note: Colors are z-score normalized to depict relative values within rows. They cannot be used to compare values between rows.

Legend

1. Brain (2) 2. Cerebellum (1) 3. Glioblastoma (30)

Proc Natl Acad Sci U S A 2005/04/19

38 samples

mRNA

9,957 measured genes

Platform not pre-defined in Oncomine

Comparison of Selected Genes in Bredel Brain 2

Over-expression in Glioblastoma vs. Normal

Rank	P-value	Fold Change	Gene	Reporter	Gene
789	9.35E-6	2.94	CDH6	IMAGE:739155	CDH6
2176	0.002	1.55	CDH2	IMAGE:325182	CDH2
2832	0.008	1.72	CDH5	IMAGE:69672	CDH5
3421	0.018	2.24	CDH11	IMAGE:251685	CDH11
3509	0.020	1.41	CDH3	IMAGE:773301	CDH3
4067	0.037	1.38	CDH24	IMAGE:155799	CDH24
5854	0.141	1.43	CDH16	IMAGE:1535734	CDH16
7002	0.260	1.12	CDH8	IMAGE:40751	CDH8
7261	0.296	1.17	CHD1	IMAGE:262996	CHD1
7711	0.364	1.10	CDH15	IMAGE:2168284	CDH15
7880	0.390	1.04	CDH17	IMAGE:511909	CDH17
8868	0.565	-1.04	CDH4	IMAGE:32777	CDH4
9561	0.682	-1.06	CDH19	IMAGE:266347	CDH19
11716	0.954	-1.39	CDH22	IMAGE:2273326	CDH22
11838	0.961	-1.89	CDH12	IMAGE:2366057	CDH12
13613	1.000	-5.07	CDH18	IMAGE:1469249	CDH18
14306	1.000	-3.58	CDH13	IMAGE:633045	CDH13
-	-	-	CDH10	-	CDH10
-	-	-	CDH20	-	CDH20
-	-	-	CDH23	-	CDH23

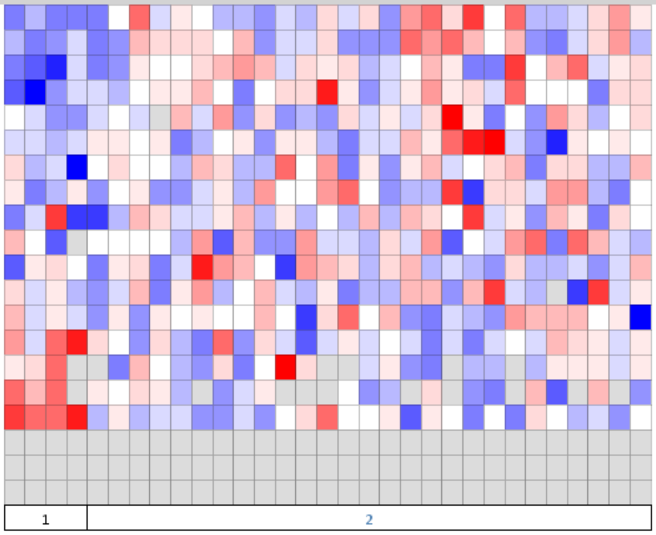

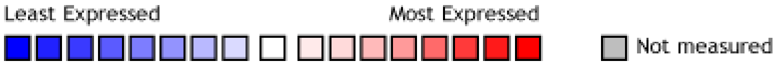

Note: Colors are z-score normalized to depict relative values within rows. They cannot be used to compare values between rows.

Legend

1. Brain (4) 2. Glioblastoma (27)

Cancer Res 2005/10/01

54 samples

mRNA

14,836 measured genes

Platform not pre-defined in Oncomine

Comparison of Selected Genes in Liang Brain

Over-expression in Glioblastoma vs. Normal

Rank	P-value	Fold Change	Gene	Reporter	Gene
586	0.005	3.63	CDH11	H96738	CDH11
724	0.009	1.96	CDH2	W48793	CDH2
1467	0.040	2.56	CDH13	AA160651	CDH13
2262	0.086	1.64	CDH6	AA421819	CDH6
2426	0.098	1.25	CDH5	T53625	CDH5
2813	0.130	1.20	CHD1	N56947	CHD1
3782	0.228	1.10	CDH8	R56219	CDH8
4481	0.326	1.07	CDH3	AA425217	CDH3
7809	0.857	-1.25	CDH17	AA088861	CDH17
7906	0.867	-1.16	CDH24	AI668564	CDH24
8522	0.923	-1.29	CDH19	N26740	CDH19
9701	1.000	-1.35	CDH16	AA919087	CDH16
-	-	-	CDH10	-	CDH10
-	-	-	CDH12	-	CDH12
-	-	-	CDH15	-	CDH15
-	-	-	CDH18	-	CDH18
-	-	-	CDH20	-	CDH20
-	-	-	CDH22	-	CDH22
-	-	-	CDH23	-	CDH23
-	-	-	CDH4	-	CDH4

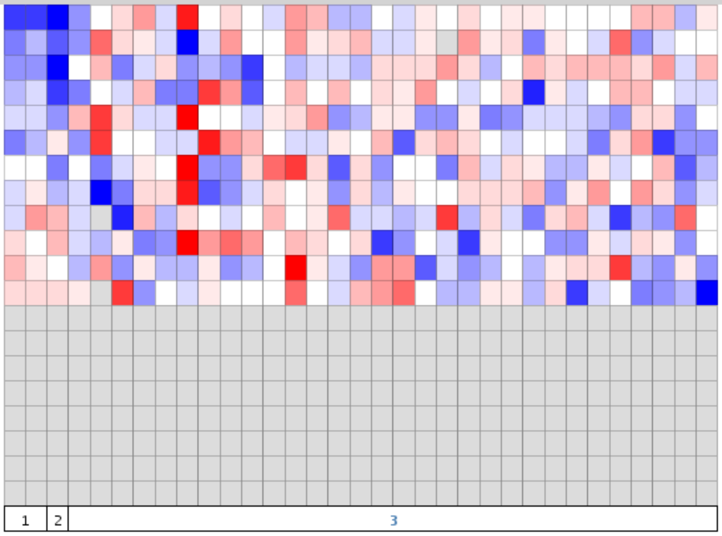

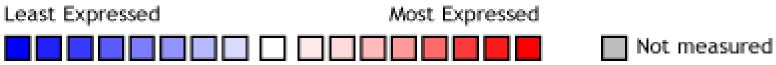

Note: Colors are z-score normalized to depict relative values within rows. They cannot be used to compare values between rows.

Legend

1. Brain (2) 2. Cerebellum (1) 3. Glioblastoma (30)

Proc Natl Acad Sci U S A 2005/04/19

mRNA

Platform not pre-defined in Oncomine

38 samples

9,957 measured genes
